# Diagnostic potential of exosomal extracellular vesicles in oncology

**DOI:** 10.1186/s12885-024-11819-4

**Published:** 2024-03-08

**Authors:** Mickensone Andre, Allen Caobi, Jana S. Miles, Arti Vashist, Marco A. Ruiz, Andrea D. Raymond

**Affiliations:** 1 Herbert Wertheim College of Medicine at, Department of Immunology and Nanomedicine, Florida International University, Miami, 33199 FL USA; 2 Medical Oncology, Baptist Health Miami Cancer Institute, Miami, 33176 FL USA

**Keywords:** Extracellular vesicles, Biomarkers, MicroRNA, Exosomes, Cancer, Daignostics, Therapeutics

## Abstract

Liquid biopsy can detect circulating cancer cells or tumor cell-derived DNA at various stages of cancer. The fluid from these biopsies contains extracellular vesicles (EVs), such as apoptotic bodies, microvesicles, exomeres, and exosomes. Exosomes contain proteins and nucleic acids (DNA/RNA) that can modify the microenvironment and promote cancer progression, playing significant roles in cancer pathology. Clinically, the proteins and nucleic acids within the exosomes from liquid biopsies can be biomarkers for the detection and prognosis of cancer. We review EVs protein and miRNA biomarkers identified for select cancers, specifically melanoma, glioma, breast, pancreatic, hepatic, cervical, prostate colon, and some hematological malignancies. Overall, this review demonstrates that EV biomolecules have great potential to expand the diagnostic and prognostic biomarkers used in Oncology; ultimately, EVs could lead to earlier detection and novel therapeutic targets.

**Clinical implications**

EVs represent a new paradigm in cancer diagnostics and therapeutics. The potential use of exosomal contents as biomarkers for diagnostic and prognostic indicators may facilitate cancer management. Non-invasive liquid biopsy is helpful, especially when the tumor is difficult to reach, such as in pancreatic adenocarcinoma. Moreover, another advantage of using minimally invasive liquid biopsy is that monitoring becomes more manageable. Identifying tumor-derived exosomal proteins and microRNAs would allow a more personalized approach to detecting cancer and improving treatment.

## Introduction

**Fig. 1:**
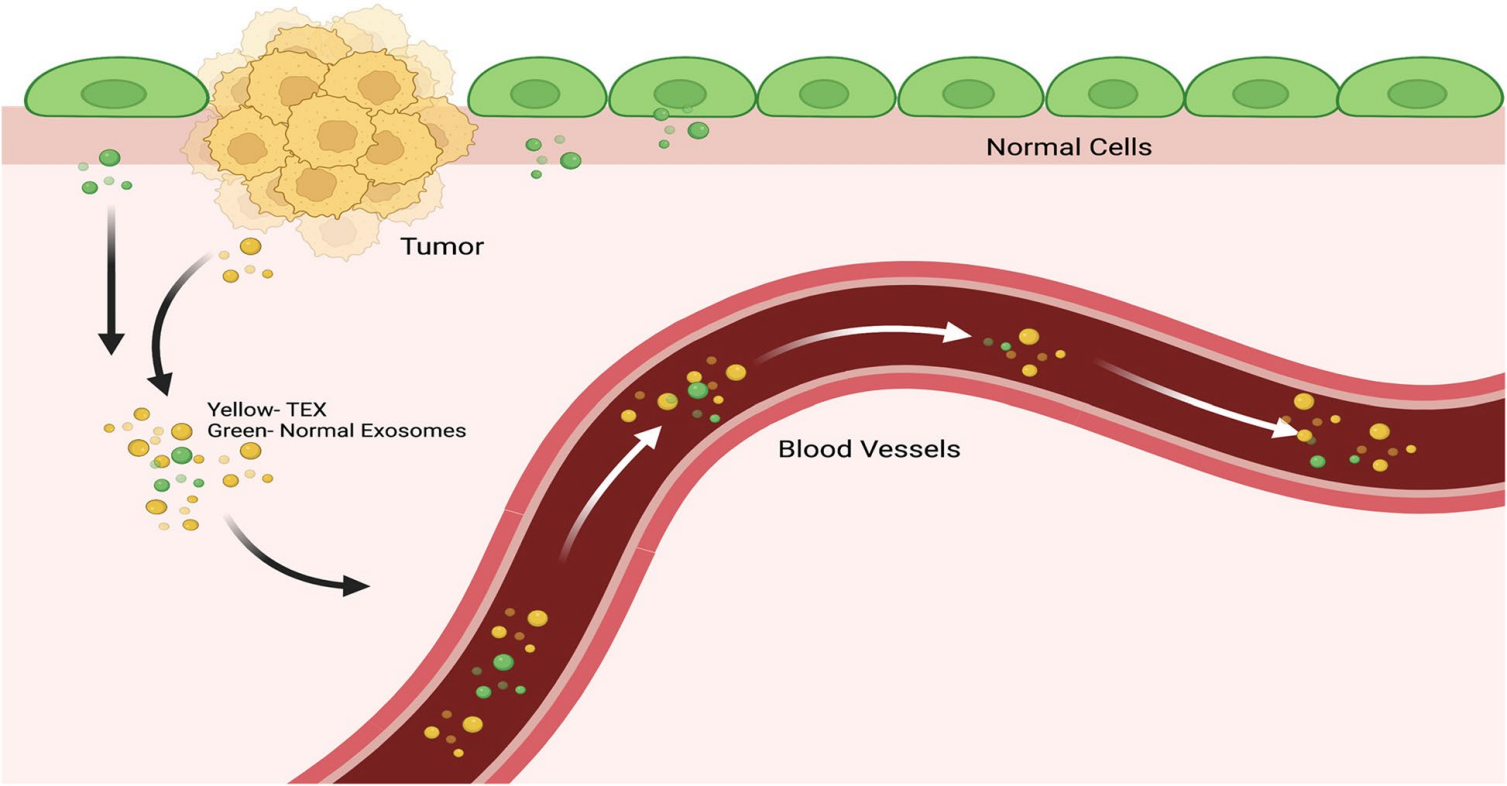
Tumor-derived exosomes (TEX) migration into the bloodstream. Cancer cells release large quantities of exosomes. In the extracellular environment, TEXs outnumber their normal exosome counterparts. These TEXs can be detected in the bloodstream or other bodily fluids. Images were created with Biorender.com and Adobe Photoshop

A liquid biopsy is a non-invasive approach to identify circulating tumor cells or tumor-cell-derived-DNA in bodily fluids such as blood, urine, saliva, and cerebrospinal fluid (CSF). Liquid biopsy has been used for early detection of cancer, responsiveness to treatment, and detection of cancer remission/re-occurrence [1]. Among the contents in a liquid biopsy, included are tumor DNA and extracellular vesicles (EVs) released by both healthy and tumor cells. Exosomes are a subtype of EVs released from most cell types into various bodily fluids, including urine, saliva, breast milk, and blood [2–4]. Exosomes tend to be near 30–150 nm in diameter and possess a cholesterol-rich lipid bilayer membrane that encloses biomolecules such as RNAs and proteins (Fig. 1). Exosomes have many functions, such as the transportation of biomolecules and cell-to-cell communication that can elicit functional responses in recipient cells that uptake them [2, 5–7]. Tumor-derived exosomes (TEXs) have multiple roles in cancer progression, and mounting evidence suggests that exosomes may also have clinical therapeutic relevance [1, 8–10]. These observations are all due in part to the contents of the exosome. Exosomes released by a cell are essentially a snapshot of the cellular status; therefore, theoretically, the cargos in an exosome derived from a cancerous/unhealthy cell are significantly different. Cancer, microbial infection, and pharmacological drugs can alter exosomal-associated cargo [11, 12]. Given this property, not only can exosome cargo serve as biomarkers for cancer but can also be utilized to monitor the effectiveness of cancer therapeutics [1, 8–10, 13, 14].

Here, we review studies on exosomal oncology as biomarkers for detection and prognosis for selected cancers. We will be focusing on proteins and RNAs because these are the most studied exosomal biomarkers, although many other types of biomarkers within exosomes such as glycan can be utilized. We will focus on the clinical potential of these exosomal biomarkers to detect skin, brain, breast, lung, liver, pancreatic, colorectal, prostate, cervical, and hematological cancer.

**Fig. 2:**
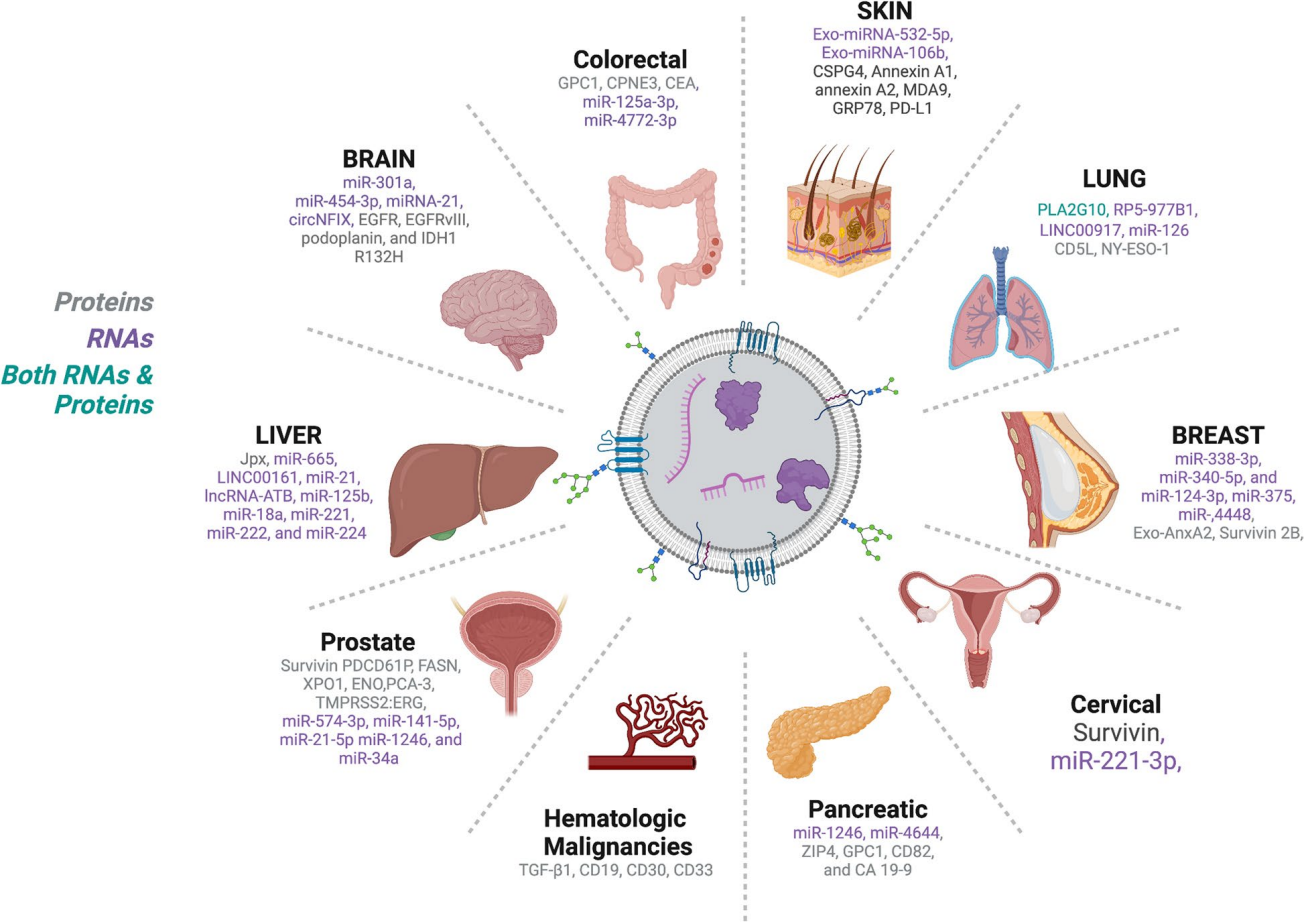
TEX Biomarkers and associated cancers: Listed are the protein and RNA biomarkers. Images were created with Biorender.com and Adobe Photoshop

Liquid biopsy utilizes bodily fluids, primarily blood, saliva, or urine to detect tumor-associated DNA, RNA, or proteins. EVs are integral components of liquid biopsies. In the tumor microenvironment, TEXs promote angiogenesis, metastasis, and evasion of immune surveillance [15–17]. Consequently, the cargos of TEXs differ significantly from exosomes from healthy non-cancerous cells. TEXs may possess biomarkers that might detect early cancer and be used for prognosis. Moreover, TEXs are more advantageous to utilize than ctDNA because exosomes possess a longer half-life and are more abundant as a biomarker than ctDNA [18]. Protein and RNA biomarkers within TEXs for different cancers are listed in (Fig. 2) and described below.

### EV biomarkers of cancers

- A. Skin Cancer (Melanoma)

Melanoma, squamous cell carcinoma, and basal cell carcinoma are the most prevalent skin cancers. Melanoma is the least frequent type in the group; however, it has a high metastatic potential and is deemed the most detrimental skin cancer in patients [19, 20]. Identifying early biomarkers of melanoma can significantly decrease mortality. However, TEXs obtained from melanoma patients can be heterogeneously mixed with non-malignant exosomes (NME) [21]. Hence, melanoma biomarkers from TEXs need to be significantly expressed to differentiate from NMEs.

One potential biomarker for melanoma is a cell surface proteoglycan called chondroitin sulfate proteoglycan 4 (CSPG4) [22–26]. In a study by Monika et al., CSPG4 in TEXs was upregulated 19-fold in patients with melanoma compared to the healthy control [27]. Another group separated TEXs from NME via a novel CSPG4-based immunoaffinity method. The ratio of TEXs to NME was 20–60% in melanoma patients [25]. Annexin A1 and annexin A2 are other potential protein biomarkers essential for melanoma invasion by signaling proliferation. TEXs from malignant melanoma cells are highly expressed with annexin A1; however, annexin A2 expression was downregulated [21]. Melanoma differentiation-associated gene 9 (MDA9) and glucose-regulated protein 78 (GRP78) are other protein biomarkers evaluated. MDA9 and GRP78 are involved in cancer invasion. TEXs from the serum of patients with metastatic melanoma expressed higher MDA-9 and GRP78 levels, indicating that these biomarkers can be used for diagnostic and prognostic testing [28]. In terms of prognosis, programmed death-ligand (PD-L1) is expressed on cancer cells more and can lead to inhibition of T cell activation. PD-L1 has also been more elevated (642-fold high) in exosomes than PD-L1 found in the blood. The test's sensitivity was 83%, and the positive predictive value was 91%. The results indicate the potential of using exosomes to monitor patients' responsiveness to therapy [29]. In addition, 60% of patients who are receiving anti-PD-1/PD-L1 therapy develop resistance to the treatment. Serratì et al. found that a high level of extracellular vesicles with PD-1 correlated with resistance to the PD-1/PD-LI therapy. By analyzing the extracellular vesicle content of patients, physicians can obtain valuable insights into their conditions and determine the most effective treatment plans. This approach can greatly enhance the accuracy of diagnoses and lead to better health outcomes [30].

Concerning RNAs, serum exosomal miRNAs such as Exo-miRNA-532-5p and Exo-miRNA-106b are more expressed in melanoma patients than in healthy individuals. These exo-miRNAs can serve as melanoma biomarkers to distinguish patients' early-stage melanoma from late-stage melanoma. Furthermore, in the study, samples from 25 melanoma patients and 25 healthy individuals, this panel of miRNAs identified 23/25 patients with melanoma (92.0% sensitivity) and 22/25 healthy individuals (88.0% sensitivity) [31]. These biomarkers indicate that exosomes can be a potent asset in melanoma detection, prognosis, and therapy.

- B. Brain Cancer (Gliomas)

Most brain cancer cases are glioma, which comprises astrocytoma, ependymoma, oligodendroglioma, and glioblastoma multiforme (GBM) [32]. GBM is the most malignant glioma. The median survival was about 15 months in one study, even with chemotherapeutic and radiological interventions [33]. Moreover, TEXs from GBM promote tumor growth and can serve as a potential biomarker [34].

In terms of prognosis, brain cancer treatment is challenging to treat due to a lack of efficient diagnostic and prognostic tools. Temozolomide (TMZ), a mutagenic agent promoting tumor death, is the standard chemotherapeutic agent for treating glioblastoma [35, 36]. However, an aggressive form of glioblastoma can become resistant to TMZ. Exosomes from TMZ-resistance glioblastoma contain upregulated circRNA nuclear factor I X (circNFIX). CircNFIX can predict the prognosis and serve as a therapeutic target [37]. The depletion of CircNFIX can enhance TMZ sensitivity. Although these biomarkers are identifiable, a highly sensitive detection tool will be needed to implement these findings in clinical settings. Shao et al. developed a rapid microfluidic chip to differentiate TEXs derived from GBM from NME [38]. They reveal that TEXs' proteins such as EGFR, EGFRvIII (a highly oncogenic variant), podoplanin, and IDH1 R132H were all highly expressed compared to healthy patients. Nevertheless, the downregulation of specific biomolecules can also serve as a biomarker. Garcia et al. reported that the expression of cytokines (IFN-γ, IL-10, and IL-13) and checkpoint molecules (CD80, CD86, and ICOS) were reduced compared to the healthy patient. Unlike PD-L1 in skin cancer, the study has found no differences between PD-L1 levels in glioma patients and the control [39].

When examining exosomal microRNA as a biomarker, miR-301a was significantly upregulated and had an AUC of 0.937. However, miR-301a levels were reduced after surgical resection of the tumor, indicating that these TEX’s were derived from the tumor. The results show the potential of using exosome microRNA as biomarkers, but it is questionable if the EVs are 100 percent exosomes because the study did not mention how they remove microvesicles with their method [40]. Exosomal miR-454-3p is an immunosuppressor that inhibits glioblastoma proliferation. miR-454-3p was expressed highly in exosomes compared to the tumor tissue [41]. The miRNA was quantified via a qRT-PCR to diagnose glioma, resulting in an AUC of 0.8663. However, it is questionable whether the samples were exosomes as well because no exosome characterizations were done. When testing for exosomal miRNA-21, the AUC was 0.927, but the study did not remove microvesicles from their samples. The study also concluded that CSF was better than serum when the exosomes were isolated via Ultracentrifugation [42]. Although these results are promising, more examinations are warranted to assess these novel biomarkers fully, and a standard exosomal isolation method is needed that removes microvesicle contamination.

- C. Breast cancer

Breast cancer (BC) characterization relies on the presence or absence of receptors such as estrogen receptor (ER), human epidermal growth factor receptor 2 (HER2), progesterone receptor (PR), and triple-negative breast cancer (TNBC). TNBC, the most aggressive type of BCs, has a high prevalence in African-American women [43–46]. Hispanic and Caucasian women have the highest prevalence of HER2 + and ER + /PR + breast cancer, respectively [43, 47–50].

Exosomal proteins, found in BC liquid biopsies, have been used for diagnostic and prognostic biomarkers applications [51, 52]. For example, exosomal annexin A2 protein (Exo-AnxA2) was elevated twofold higher in serum-derived exosomes in African American women with TNBC than in the healthy control and correlated with the tumor grade. Compared to the healthy control, patients with TNBC, ER + , and HER2 + had Exo-AnxA2’s AUC values of 1, 0.83, and 0.99, respectively [53]. Moreover, exosomal Survivin 2B, an anti-apoptotic protein elevated in BC patients, was able to detect early-stage BC [54]. While the exosomal protein is CD24; it was found in serum-derived exosomes and indicated the late stage of BC [55].

Exosomal nucleic acid content can differentiate molecular signatures of BC from non-cancerous cells [51, 56–58]. Patients with recurrence BC were found to have elevated miR-338-3p, miR-340-5p, and miR-124-3p and downregulated miR-29b-3p, miR-20b-5p, miR-17-5p, miR-130a-3p, miR-18a-5p, miR-195-5p, miR-486-5p, and miR-93-5p in serum-derived TEX [59]. Moreover, serum exosomes of ER + BC patients had elevated levels of miR-375 (AUC of 0.96), miR-221, miR-210, and miR-10b compared to healthy donors as detected by thermophoretic sensor implemented with nanoflares (TSN). The technique is novel and does not require RNA extraction [60]. Exosomal miRNA profiles, specifically miR-,4448 miR-2392, miR-2467-3p, and miR-4000-3p, can be used to determine the efficacy of TNBC treatment. The AUC was 0.7652 [61]. Together, these findings show that exosome cargo in BC liquid biopsies, serum, or plasma are effectively used as biomarkers for cancer detection, staging, and prognosis.

- D. Lung and Bronchus

The leading cause of death in cancer patients is lung/bronchus cancer (LC) and it is difficult to diagnose. It is estimated that 85% of cancers are non-small cell lung cancer [62]. About 75% of lung cancer patients get diagnosed at stage 4 when the cancer has metastasized [63, 64]. Low-dose computed tomography (LDCT) is recommended for LC screening but needs to be confirmed with surgery and mostly results in false positives. Surgical resection is usually the treatment of choice when the tumor is at an early stage. Early liquid biopsy can be beneficial to early lung cancer screening. However, biomarkers need to be identified.

One potential protein biomarker for LC is CD5L. Researchers isolated 55 upregulated proteins from TEX and CD5L was the protein with the highest AUC with a value of 0.943 [65]. NY-ESO-1 is another protein biomarker tested for LC. The exosomes were isolated via a microarray and when tested, NY-ESO-1 resulted in an inferior survival hazard rate of 1.78 after Bonferroni correction [66]. Exosomal protein PLA2G10 and its mRNA both are potential biomarkers for LC. The PLA2G10 protein resulted in an AUC of 0.859 alone while the mRNA exhibited an AUC of 0.770. When both the PLA2G10 mRNA and protein were combined as a biomarker, the AUC was 0.873 [67]. Other exosomal RNAs, such as long non-coding RNA RP5-977B1 were found to be a potential biomarker for LC. RP5-977B1 with an AUC value of 0.8899. While the finding is outstanding, the exosome isolation method utilized a precipitation-based methodology which could result in sample contamination with microvesicles. This biomarker may also be detected in other EVs. A study examining the diagnostic potential of serum-based exosomal long intergenic noncoding RNA 917 (LINC00917) in non-small cell lung cancer (NSCLC) identified LINC00917, as another potential exosomal biomarker, was with an AUC of 0.811 [68]. However, these investigators also isolated serum exosomes via precipitation allowing for possible microvesicle contamination [69]. The expression of miR-126, a microRNA known to be modulated in cancer progression, was compared in the serum, exosomes, and exosome-free serum of NSCLC patients and healthy controls [70]. For advanced stage NSCLC patients, miR-126 was down-regulated only in the serum, whereas in healthy control an equal distribution of miR-126 was found in exosomes and exosome-free serum [70]. Interestingly, miR-126 was present in exosomes of both early and advanced stage NSCLC patients, thereby suggesting that miR-126 was a potential biomarker for LC [70].

- E. Gastrointestinal cancers

### Pancreatic cancer

Pancreatic cancer (PC) was ranked as the 14th most common cancer in 2018, and the 7th highest cause of cancer mortality in the world [71]. Early detection is a key to combating PC; however, inexpensive, and rapid routine examinations have yet to be developed. The best option to treat PC is currently surgery; however, even though a survival benefit with adjuvant treatment has been demonstrated, 71%—76% percent of patients relapse within two years [71]. Thus, surgery is usually paired with chemo-radiotherapy to improve the patient survival rate [71]. Recent studies suggest that EV nucleic acids and proteins may function as biomarkers for the diagnosis of PC [72]. The most frequently reported EV RNA for the diagnosis of PC were miR-21 and miR-10b, whereas the most reported EV proteins were GPC1 and EphA2 [72].

Recently, zinc transporter protein 4(ZIP4), known to promote tumor proliferation, migration, and invasion, was upregulated in exosomes and clinical serum of PC patients with an AUC of 0.8931 compared to healthy control[73]. Glypican-1 (GPC1) is another protein enriched in TEXs released from PC cells. These GPC1 containing TEXs derived were shown to have a 100% sensitivity and specificity with an AUC of 1.0, making GPC1 an excellent diagnostic biomarker of PC[74]. This finding was controversial because Frampton et al. also assessed the diagnostic potential of exosomal GPC1 in PC and found that GPC1 only had an AUC of 0.59 and a sensitivity and specificity of 74% and 44%, respectively [75]. The inconsistency of these results may be attributed to the sample size, exosome isolation method, and the assay used to detect GPC1. Another study demonstrated that rather than GPC1 protein, miRNAs specifically miR-10b, miR-21, miR-30c, miR-181a, and miR-let7a) serve as better biomarkers of PC [76]. The differences in their results could be due to the differences in centrifugation speeds used in their isolations. Unlike the other group, Lai et al. [75], pre-cleared samples using 10,000 g. New studies on EV isolation, have recommended centrifuging samples at a minimum of 20,000 g for 30 min to remove microvesicles [77, 78]. Xiao et al. developed a standardized method to re-assess results obtained by Melo et al. and obtained an AUC of 0.885 for exosomal GPC1. However, coupling GPC1 with an exosomal cluster of differentiation 82 (CD82) and carbohydrate antigen 19–9 (CA 19–9) resulted in an AUC of 0.942, showing that the method improved the identification PC biomarkers in the Chinese cohort[79]. Within these studies, the isolation of exosomes and detection of the biomarker methods differ from each other. A consistent method is warranted to assess these biomarkers systematically. Using a murine model of PC, investigators recently reported that saliva-derived TEXs could be utilized to detect early-stage PC [80]. Machida et al. conducted a pilot study that examined saliva-derived TEXs of patients with pancreaticobiliary tract cancer and found miR‑1246 and miR‑4644 as potential biomarkers. The AUC for miR‑1246 and miR‑4644, combined was 0.814, and individually 0.763, and 0.833, respectively [81]. A similar study by Xu et al., found miR-196a and miR-1246 to be highly enriched in TEXs from pancreatic cancer patients with an AUC of 0.81 and 0.73, respectively [82]. Although these studies had a small sample size, the results showed the great potential for non-invasive PC diagnosis using exosomal biomarkers.

### Liver cancer

In adults, one of the common types of liver cancer is Hepatocellular Carcinoma (HCC). Intrahepatic cholangiocarcinoma (ICC) accounts for the other liver cancer subtypes [83]. Sub-Saharan Africa and Southeast Asia have a greater liver cancer prevalence than the United States [83–85]. There are about 800,000 new cases each year [85]. Moreover, about 700,000 deaths annually make liver cancer a leading cause of cancer-related death worldwide, according to the American Cancer Society. Current treatments for liver cancer remain poor, and the 5-year survival rate for liver cancer patients with or without a liver transplant is 60–70% or 33%, respectively [86, 87]. Early diagnosis can help decrease the mortality rate; however, the current HCC detection method yields a low sensitivity of only 60% [88].

Currently, surveillance for HCC is determined by the plasma marker alpha-fetoprotein (AFP) and ultrasonography [89]. However, AFP is not sensitive enough for early diagnosis or staging because the protein is consistently elevated in 45% of the cases. Circulating Xist expression in the peripheral blood is a more sensitive indicator than AFP and may assist as an early diagnostic indicator of HCC in females. Nevertheless, recent reports suggest that lncRNA X inactivates specific transcript (Xist) and is only elevated in the whole blood of female patients with HCC [90]. Exosomal Jpx allows the contrast between early-stage HCC, healthy controls, CHB, LC, and AFP in female HCC patients. Exosomal Jpx could therefore be a potential biomarker [90].

Several other predictive and prognostic exosomal miRNA biomarkers have been recognized for HCC and may guide treatment strategies [91]. A direct correlation between the tumor size and exosomal miR-665 level was seen in the patient serum [92]; moreover, high exosomal miR-665 reflected decreased prognosis/survival time in the HCC group. A similar trend was seen for RNA LINC00161 in urine-derived exosomes of HCC patients compared to healthy controls. The study yields an AUC of 0.794 with sensitivity and specificities of 75.0% and 73.2%, respectively [93]. miR-21 was more enriched in exosomes from HCC patients than in exosomes of chronic hepatitis B (CHB) patients and healthy controls [94]. Regarding prognosis, miRNA-21 and lncRNA-ATB correlated with HCC tumor size, spread to the lymph nodes, and metastasis to other body parts [95]. In another study, patients with liver cirrhosis, chronic hepatitis B, and HCC were found to have upregulated exosomal miR-125b. Compared to the CHB and LC groups, the miR-125b level was lower in the HCC cohort and correlated with the number of tumors and TNM stage [96]. Exosomal circPTGR1 elevated in HCC patients correlated with metastasis, serving as a biomarker for HCC prognosis and clinical staging [97]. The levels of miR-18a, miR-221, miR-222, and miR-224 were heightened in subjects with HCC compared to subjects with Hepatitis B (HBV), thereby making these three miRNAs also biomarkers of HCC [98]. Elevated expression of miR-92a was shown to promote proliferation and block apoptosis of HCC-derived [99, 100]. Low miR-92a expression, compared to healthy donors, has been recorded in HCC patients [99]. Furthermore, when miR-92a was downregulated, tumor growth was suppressed in tumor-bearing nude mice [100]. This suggests that the dysregulation of miR-92a can indicate the development of HCC. As reviewed here, exosomes have the potential to be an inexpensive, non-invasive tool for early diagnosis, staging, and prognosis of HCC.

### Colorectal cancer

Approximately 50% of colorectal cancer (CRC) patients are diagnosed with stage 2 or stage 3 cancer [101]. Early testing has reduced the CRC mortality rate. For example, a colonoscopy could be employed to identify tumors; nonetheless, these tests are typically not performed during a regularly scheduled physical examination [102]. Thus, there is a need for a quick and straightforward CRC diagnostic test. Blood-derived circulating exosomes could provide one such test, as they may serve as biomarkers identifying tumor cells. Hence, the relationship between TEXs and colorectal tumors has been the focus of multiple studies thus far.

As mentioned above, exosomal GPC1 has been studied as a biomarker for PC. It has been studied as a biomarker for CRC as well. For example, GPC1 is upregulated in plasma-derived exosomes in patients with colorectal cancer [103]. Copine III (CPNE3) is a calcium‐dependent membrane‐binding protein and is upregulated in prostate, ovarian, and breast cancer patients, and CPNE3 has been found in exosomes. When tested to detect CRC, CPNE3 yielded an AUC of 79.1% alone and 83% when combined with Carcinoembryonic antigen (CEA), a known CRC marker[104].

Wang et al. demonstrated that miR-125a-3p was significantly upregulated in early-stage CRC patient plasma exosomes. miR-125a-3p was measured alongside CAE; the AUC was increased to 85.5%, thus demonstrating the capacity of miR-125a-3p as an early-stage CRC biomarker [102]. Levels of miR-4772-3p in serum exosomes may be a prognostic tool in predicting stage 2 and 3 colon cancer recurrence [101]. Liu et al. found that there was a significantly lower level of baseline exosomal miR-4772-3p in 27 patients with recurrent disease relative to the levels observed in the 57 patients without recurrence. The recurrence time was 5.48-fold shorter in patients with a decreased baseline expression of miR-4772-3p; an elevated risk of death of 6.19-fold was also found in these patients. Given this data and the fact that the biomarker was readily measurable at baseline levels within the circulating blood, miR-4772-3p may potentially serve as a biomarker capable of identifying tumor recurrence in stage II/III colon cancer patients. Therapeutic and diagnostic strategies employing exosomes are still in the early stages of development and implementation. Thus, further studies are necessary before the validation of exosomes as therapeutic, diagnostic, or prognostic.

- F. Reproductive Cancers

### Cervical cancer

Cervical cancer (CC) is a common cancer affecting women. The mortality rate for CC in developed nations is low; additionally, treatments and screening options in developed nations are available. However, early predictive diagnosis is required to maintain a national low mortality rate. Recent studies suggest that vaginal secretion or serum-derived exosomes may serve as potential biomarkers for the diagnosis of CC [105]. TEXs from CC have a significant role in the progression of CC and its phenotypic aggressiveness [106, 107].

Survivin is highly expressed in CC cells. Survivin has been observed to be highly concentrated within exosomes released from CC cells [108, 109]. Another CC biomarker includes exosomal miR-221-3p, which down-regulates mitogen-activated protein kinase (MAPK10), hence promoting metastatic potential [107]. exosomes containing activating transcription factor 1 (ATF1) and RAS genes are also potential biomarkers of CC. These exosomal cargos were upregulated in a humanized tumor mouse model of CC, thus functioning as potential CC biomarkers [110]. Additionally, if a biopsy is acquired, studies have shown that PI3/Akt/mTOR gene expression is upregulated in histopathological tissues of CC, providing yet another possible biomarker of CC [105]. The biopsy is both painful and invasive, whereas it is a superior diagnostic marker as it requires a less invasive or painful means of acquisition. For example, it has been reported that vaginal lavage-derived exosomes contain levels of PI3/Akt/mTOR gene expression comparable to biopsied histopathological tissues [105]. Survivin, miR-221-3p, ATF1, RAS, and PI3/Akt/mTOR expression are all upregulated in CC cells and are exosomal constituents. These serve as potential therapeutic targets for CC treatment in addition to biomarkers.

### Prostate cancer

Prostate cancer (PCa) is the fifth leading cause of cancer-related deaths among men and is the most common cancer in men globally [111]. In the United States, PCa is the second leading cause of cancer-related deaths in men [112]. PCa can either remain latent or progress slowly or aggressively [113, 114]. Prostate-specific antigen (PSA) and Digital rectal examination (DRE) are the primary clinical diagnostic protein biomarkers and prognostic indicators of PCa, respectively. Measuring PSA levels in the blood may help indicate the presence of prostate cancer, especially if the levels are high. However, the PSA test lacks specificity and has been demonstrated to have a high probability of false positives [115, 116]. New biomarkers are needed to prevent unnecessary invasive procedures, such as prostate biopsies, and to support an optimized and accurate treatment plan. Prostate cell-derived exosomes may contain several potential biomarkers [117].

Proteomic profiling of exosomes derived from prostate cell lines PC346C and VCaP identified a few potential novel candidate biomarkers of PCa, including PDCD61P, FASN, XPO1, and ENO, which are all elevated [118]. Plasma-derived exosomal Survivin is another potential biomarker for early detection of PCa [119]. Similar to cervical cancer, elevated levels of Survivin were observed in PCa patients relative to healthy controls and patients with benign prostate hyperplasia (BPH). Additionally, exosomal Survivin level was also greater in PCa patients who relapsed on chemotherapy, suggesting that exosomal Survivin could be an indicator of therapeutic responses [119]. However, Survivin did not correlate with the Gleason grading system used to estimate the prognosis of men with PCa, suggesting that multiple PCa biomarkers may be needed [119, 120]

Aside from exosomal proteins, several RNAs and miRNAs have been detected in urinary exosomes of PCa patients, including PCA-3, TMPRSS2:ERG [121], and five down-regulated miRNAs – miR-34a-5p, miR-143-3p, miR-196a, miR-501-3p, and miR-921–1-5p [122]. Urinary exosomal miRNAs such as miR-574-3p, miR-141-5p, and miR-21-5p were found to be elevated in PCa patients compared to healthy people with an AUC of 0.85, 0.86, and 0.65 respectively [123]. Recently, miR-1246, a blood-derived exosomal miRNA, has been identified as a potential PCa biomarker. The researchers concluded that miR-1246 might be a great predictor of aggressive prostate cancer, resulting in an AUC of 0.926, 100% specificity, and 75% sensitivity [124]. Lastly, prostate cancer cell-derived miR-34a regulates multiple RNA targets that have been shown to possess a strong relationship with castration-resistant prostate cancer (CRPC) prognosis and progression [125]. Docetaxel is often the first line of treatment against CRPC; however, there is innate and acquired resistance to CRPC. The downregulation of miR-34a could also indicate a potential early treatment failure. Altogether, these findings demonstrate that serum/urine-derived exosome cargo may be employed to replace PSA as a biomarker of PCa.

- G. Hematologic Malignancies

Hematologic malignancies (HMs), such as leukemias, are among the most common forms of cancer in the United States, with over 60,000 new cases annually [126]. HMs originate from cells in the blood and are classified according to their lineage of origin, which include lymphoid/lymphoblastic, myeloid/myelogenous, or histiocytic/dendritic neoplasms. Among the various types of leukemias, the prognosis varies and is dependent on molecular, genetic, and clinical characteristics of the specific neoplasm; for example, acute myelogenous leukemia (AML) has the poorest prognosis with a 25% five-year survival rate. Patients with HMs have higher levels of TEXs relative to matched controls. NK cell activity is lowered by TEX, which also suppresses the expression of NGK2D, resulting in the disruption of the normal immune response to HMs and predisposing individuals to relapse [127]. TEX-induced immunosuppression may inhibit cell differentiation, apoptosis of cytotoxic T cells, and tolerance development [128, 129]. Therefore, TEX may function in prognosis estimation by forecasting HM relapses. Within the TEX of AML patients, specific proteins, such as TGF-β1, have been recorded at high levels and correlate with the chemotherapy response [130].

Given that the transfer of RNA to other cells is a crucial mechanism of tumorigenesis, both the quantity and composition of TEX in HM patients may influence disease progression and act as a marker to assess therapeutic efficacy [131, 132]. Plasma levels of EVs were raised significantly relative to matched controls for HMs such as chronic lymphoblastic leukemia (CLL), AML, non-Hodgkin's lymphomas, and Hodgkin's lymphomas (HL) [132]. Other components, such as specific RNAs, FLT3-ITD, NPM1, IGF-IR, and CXCR4, have impacted the prognosis and treatment response [133, 134]. Tumor-associated antigens (TAAs) such as CD19 in B-cell malignancies, CD30 in HL, and CD33 have been detected in EV released by myeloid neoplasms [132]. The molecular content of leukemia-associated TEX differs from exosomes derived from the normal hematologic physiology [135–137]. For example, EV levels in AML patients contain leukemia-associated antigens (LAA) and markers of myeloblastic activity [130]. Furthermore, in patients with non-solid tumor HMs, there are increased levels of clotting factor-rich TEX, suggestive of a graver prognosis [130, 138]. The growing understanding of TEX-mediated molecular, neoplastic, and malignant processes involved in HMs implicates TEX as a potential therapeutic target for advancing leukemias' diagnosis, treatment, and prognosis-estimation accuracy.

- H. Translational development

**Fig. 3:**
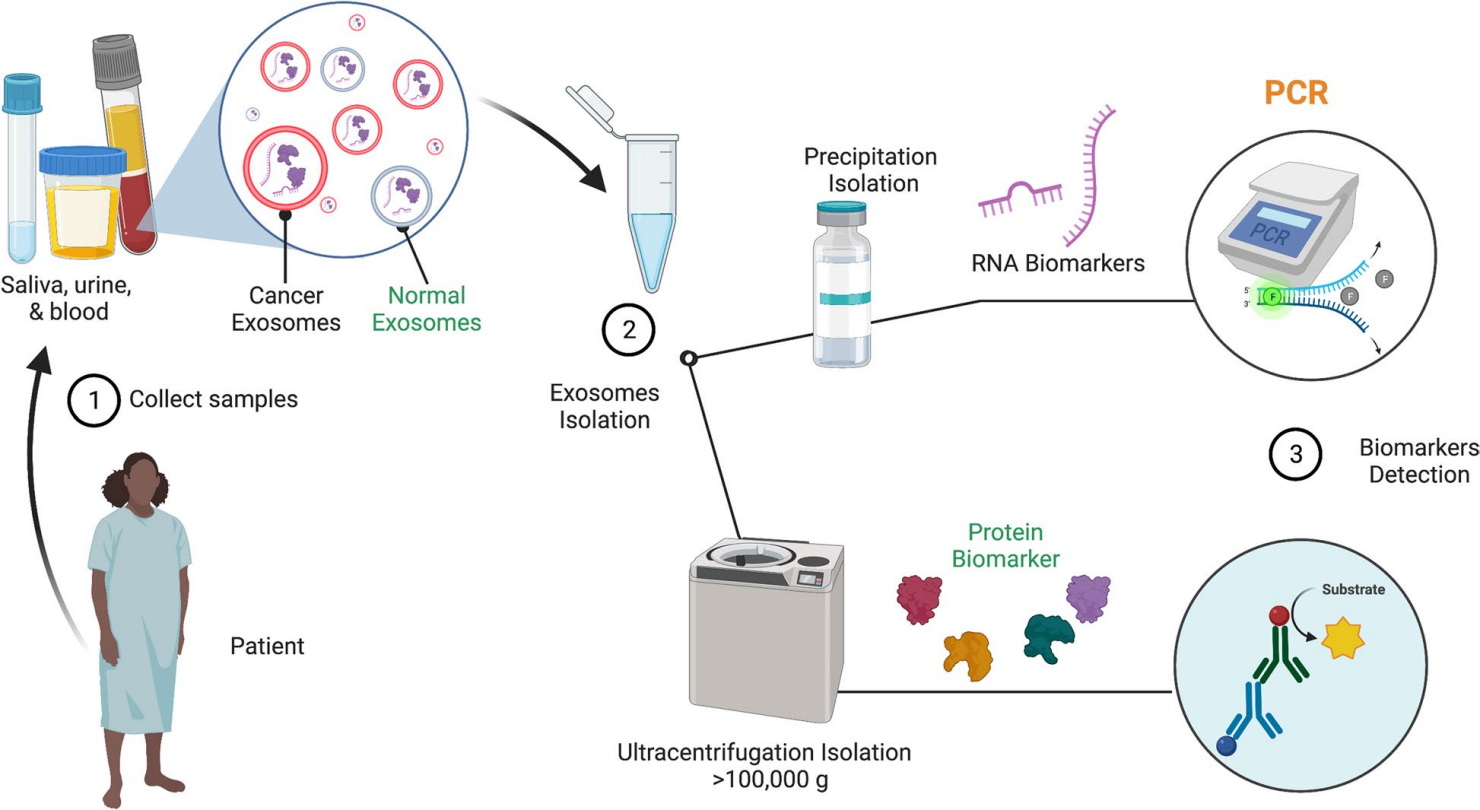
Methods used to isolate TEX and detect their biomarkers in personalized medicine. Step 1—collect patient biofluid sample such as saliva, urine, or blood. Step 2—isolate exosomes from these samples via a variety of methods. Step 3—detect TEX with varying methods such as mass spectrometry, ELISAs, or western blots for proteins, PCR or RNAseq for RNA, and multiplexed microarrays all of which are isolation-dependent. The pairing of techniques can be key for the optimization of biomarker detection. Images were created with Biorender.com and Adobe Photoshop

The methods used are critical for translating a particular biomarker to the clinical setting. An overview of the methods used to identify biomarkers within TEXs is depicted below in Fig. 3. For example, large sample sizes used in clinical trials may require high-throughput methods for isolating EVs. Current techniques such as differential centrifugation are time-consuming and have a low exosome yield. Precipitation and chromatography methods isolating exosomes can be expensive. Methods allowing for increased EV purity, high-yielding, rapid, and economical exosomal EV isolation can help expedite the transition of these exosomal biomarkers to clinical use. Most of these studies precleared their samples at centrifugal speeds below 10,000 g. This speed is ineffective for removing small microvesicles. A centrifugation speed of approximately 20,000 g is required to pellet most microvesicles [78, 139–142]. It is impossible to conclude that these results are due to exosomes due to the lack of EV characterization. Therefore, it is best to interpret these results as coming from the complete subset of EVs.

There are various methods for isolating exosomes, each with advantages and disadvantages. The ultracentrigation (UC) method is the gold standard for isolating exosomes through high centrifugation. It results in the purest exosome EV preparation; however, only 10–20% of exosomes are separated from the sample [69, 143]. Precipitation of exosomes using polymers such as polyethylene glycol results in high exosome recovery; however, it is the most contaminated method. [144]. IC method isolates exosomes using antibody-conjugated beads targeting CD63, CD9, and CD81 surface proteins. IC can isolate subpopulations of exosomes, which is one of the advantages of implementing this technique [145]. The disadvantage is that this method is expensive and time-consuming. In SEC (Size Exclusion Chromatography), the sample is introduced into a mobile phase that passes through a porous stationary phase[146]. During this process, larger particles will have a shorter retention time and come out first, while smaller particles will have a longer retention time and come out last. Exosomes isolated via SEC have high recovery and purity; however, SEC works with small sample volumes and can be expensive to obtain. Microfluidic-based isolation is a method to isolate exosomes with a device/chip that separates exosomes in various ways, such as immunoaffinity, size, and density [147]. The technique is faster and has high purity. However, microfluidics can be expensive. Overall, the EV isolation method determines EV purity and subsets. The type of method to isolate exosomes is essential to achieving the best area under the curve (AUC). For example, exosomal RNA biomarkers may have a high AUC when the TEXs are isolated via the precipitation method. Although higher exosome yield can be obtained via precipitation, the samples are often contaminated with microvesicles and the reagent solution (i.e. polymer) used in the process [139–141]; thus, making the type of EV containing the RNA biomarker difficult to ascertain. In contrast, exosomal protein biomarkers yield high AUCs when the exosomes are isolated via differential centrifugation (UC). Risha et al. compared three exosomal isolation methods, Exo-quick, UC, and ultrafiltration–ultracentrifugation. For defining exosomal biomarker proteins, the UC method yields the purest preparations with a uniform exosomal size that is compatible with downstream analysis via mass spectrometry [142].

Detection of EV biomarkers are dependent upon isolation methodology and the most economical procedure resulting in the best AUC is likely to be used in the clinic. An overview of methods used to define EV (particularly exosomes) biomarkers in Oncology is outlined in Fig. 3. Detecting EV RNA biomarkers may be more ready for clinical use than protein biomarkers because most clinics already have access to a PCR; thus, clinics will save and not have to keep ordering TEXs detecting kits such as ExoDx, a commercially available exosomal test for prostate cancer. Hence it might be an advantage to detect RNA biomarkers over protein biomarkers [143].

## Conclusions

Overall, this review of biomarkers for cancers detected in EVs shows how integral EVs have become in Oncology. EV biomarkers for selected cancers are summarized below in Tables 1 and 2. These biomarkers not only serve as diagnostics or prognostic indicators but can also act as mediators of tumorigenesis and metastasis. This dichotomy reflects the great potential of EV-based biomarkers in Oncology. Understanding the content of EVs released during cancer can define biomarkers that reflect individual differences in cancer development, progression, and treatment responses. Most importantly, EV biomarkers represent non-invasive, economical means to study cancers and can also reveal potential therapeutic targets. EVs in liquid biopsies are likely to become even more integral to novel personalized approaches for detecting, monitoring, and treating cancers in the future.

**Table 1:** Summary of selected cancers and their exosomal biomarkers

Cancers	Source		Exosomal Biomarkers	Isolation Method			Observation	Reference
**Melanoma**	Antigen		Chondroitin sulfate proteoglycan 4	DC, SEC, immune capture			Malignant exosomes were separated from non-malignant exosomes	[24]
**Brain** **Glioma**	CSF		miR-301a	DC, filtration, commercial reagent			Serum exosomal miR-301a levels are significantly upregulated in human glioma patients	[39]
	Blood plasma		EGFRvIII, podoplanin, IDH1 R132H	Microfluidic device			EVs from GBM displayed increased expressions of EGFR, EGFRvIII, PDPN, and IDH1 R132H	[45]
			miR-335, miR-301a, and miR-454-3p	Commercial Reagent (ExoQuick™)			miR301 diagnostic and prognostic;miR-454 lower in post operative vs. preoperative glioma patients	[39, 40]
**Breast**	Serum		Annexin A2	Commercial Reagent			(i) Serum Exo-AnxA2 upregulated in TNBC patients	[82]
	Serum		miR-375, miR-221 miR-210 miR-10b	DC			(ii) Higher levels of miR-375, miR-221, and miR-10b were found in ER + BC patients compared to healthy donors	[59]
**Reproductive Cancers**								
**Cervical**	Epithelial cervical cancer cells		Survivin	DC			Survivin was detected on and within HeLa-derived exosomes	[106]
	Epithelial cervical cancer cells		miR-221-3p	DC			Downregulates MAPK10 expression in cervical cancer cells	[107]
	Serum		Activating transcription factor 1 RAS upregulation	Commercial Reagent			Elevated levels in cervical cancer mouse tumors	[110]
	Vaginal Secretions		PI3/Akt/mTOR upregulation	Commercial Reagent			PI3/Akt/mTOR gene expression is elevated in cervical cancer tissue	[105]
**Prostate**		Prostate cancer cells	miR-21		UC	Expression down-regulated by exosomal RNA gold nanoparticle conjugates upregulated in urinary exosomes		[148]
		Plasma	piR-349843, piR-382289, piR-158533, and hsa-piR-002468		UC	Expression of these piwi RNAs was upregulated in urinary extracellular vesicles		[149]

**Table 2:** Summary of exosomal biomarkers for selected hematological malignancies and gastrointestinal cancers and

Cancers	Source	Exosomal Biomarkers	Isolation Method	Observation	Reference
**Gastrointestinal**					
**Liver**	Serum	miR-18a, miR-221 miR-222, miR-224	Commercial Reagent	All four miRNAs were elevated in patients with HCC	[98]
	Serum	miR-665,	Commercial Reagent	Tumor size directly correlated with levels of miR-665	[92]
**Hepatocellular carcinoma** **(HCC)**	Plasma	miR-92a, miR-122	DC	Increased levels of miR-21 and miR-125b in HCC patients	[94]
				Dysregulation of miR-92 levels is indicative of HCC development	[100]
**Pancreatic**	Plasma	miR-196a miR-1246 miR-21, miR-155, and miR-31	DC, Commercial Reagent	Biomarker of localized pancreatic cancer	[82, 148]
	Saliva	miR‑1246 and miR‑4644	Total Exosome Isolation	miR‑1246 and miR‑4644, the results yielded an increased AUC of 0.833	[81]
	serum	Glypican-1	DC	Levels of GPC1 + crExos correlate with tumor burden and survival in patients pre- and post-surgical tumor resection	[74]
**Colon**	Colon cancer cells	PTEN downregulation	ExoQuick™, commercial reagent	(ii)Exosomes promote cetuximab resistance by PTEN downregulation	[149]
	Plasma of CRC patients	Copine III(CPNE3)	Commercial reagent	Increased expression colorectal cancer(CRC)	[104]
**Hematological Malignancies**					
**Acute Myelogenous Leukemia** **(AML)**	Tumor-derived exosomes (TEX)	Leukemia-associated antigens (LAA) TGF-B1	Differential Centrifugation	Increased myeloblastic activity	[130]
**B-cell malignancy** **Hodgkin's Lymphoma (HL)** **Myeloid neoplasms**	Tumor-associated antigens (TAAs)	CD19 CD30 CD33	ExoQuick™, commercial reagent	Diagnostic of prostate cancer patients	[132]

## Data Availability

Data will be available upon request. Please email the corresponding author with requests.
